# Sensitivity of a Ribavirin Resistant Mutant of Hepatitis C Virus to Other Antiviral Drugs

**DOI:** 10.1371/journal.pone.0074027

**Published:** 2013-09-05

**Authors:** Kathleen B. Mihalik, Dino A. Feigelstock

**Affiliations:** Division of Viral Products, Center for Biologics Evaluation and Research, FDA, Bethesda, Maryland, United States of America; Kobe University, Japan

## Abstract

**Background:**

While ribavirin mono-therapy regimens have minimal effect on patients with chronic hepatitis C virus (HCV) infections, they can be efficacious when combined with interferon. Clinical studies show that interferon-free combination therapies containing ribavirin are also efficacious, suggesting that an interferon-free therapy could be adopted in the near future. However, generation of drug resistant mutants and cross resistance to other drugs could impair the efficacy of the treatment. Therefore, understanding the mechanism of HCV resistance to ribavirin and cross resistance to other antiviral drugs could be of major importance.

**Methods:**

We tested the ability of a J6/JFH1 derived HCV ribavirin resistant mutant to grow in tissue cultured Huh7D cells in the presence of the mutagen 5-Fluorouracil and the nucleoside analog 2′-C-Methylcytidine. Virus replication was assessed by detecting HCV antigens by immunofluorescence and by titrating virus present in the supernatants. Recovered viruses were amplified by RT-PCR and sequenced.

**Results:**

The sensitivity of HCV-RR relative to parental J6/JFH1 to the tested drugs varied. HCV-RR was more resistant than J6/JFH1 to 5-Fluorouracil but was not more resistant than J6/JFH1 to 2′-C-Methylcytidine. Growth of HCV-RR in 5-Fluorouracil allowed the selection of an HCV-RR derived mutant resistant to 5-Fluorouracil (HCV-5FU). HCV-5FU grows to moderate levels in the presence of high concentrations of 5-Fluorouracil and to parental levels in the absence of the drug. Sequence of its genome shows that HCV-5FU accumulated multiple synonymous and non-synonymous mutations.

**Conclusions:**

These results indicate that determinants of resistance to ribavirin could also confer resistance to other anti-HCV drugs, shedding light toward understanding the mechanism of action of ribavirin and highlighting the importance of combination drug selection for HCV treatment. The results also show that it is possible to select a 5-Fluorouracil HCV resistant mutant that replicates to levels similar to parental virus when grown in the absence of 5-Fluorouracil.

## Introduction

Hepatitis C virus (HCV) is an enveloped, positive strand RNA virus member of the genus *Hepacivirus* of the *Flaviviridae* family. The HCV genome consists of an RNA molecule of approximately 9.6 kb in size containing a single open reading frame flanked by structured 5′ and 3′ un-translated regions. An internal ribosome entry site directs the translation of a polyprotein precursor that is cleaved by cellular and viral proteases into 10 proteins (core, E1, E2, p7, NS2, NS3, NS4a, NS4b, NS5a, and NS5B) (reviewed in reference [1]). Human HCV infection causes chronic liver disease, cirrhosis, and is associated with hepatocellular carcinoma [2]. It is estimated that 180 million people worldwide are infected with HCV [3] and given the chronic nature of the infection it is expected that the number of patients with hepatocellular carcinoma will increase in the coming years.

The standard therapy for the treatment of chronically HCV infected patients consists of a combination of pegylated interferon alpha and ribavirin [4]. Recently, two protease inhibitors were approved by the FDA and are being used in the clinic [5], [6]. Given the side effects associated with injections of interferon, an interferon-free regimen for the treatment of HCV infections is highly desirable. Recent studies have shown that ribavirin in combination with other antiviral drugs, without interferon, can be efficacious [7], [8], suggesting that an interferon-free therapy containing ribavirin could be adopted in the near future. However, generation of drug resistant mutants and cross resistance to different drugs could impair the efficacy of the treatment. In addition, the anti HCV mechanism of action of ribavirin is not completely elucidated. Several mechanisms of action of ribavirin against HCV were proposed including a direct effect against the HCV RNA dependent RNA polymerase (NS5b); induction of misincorporation of nucleotides leading to lethal mutagenesis; depletion of intracellular guanosine triphosphate pools; alteration in the cytokine balance from a Th2 profile to a Th1 profile; and up-regulation of genes involved in interferon signaling [9], [10].

In order to study the cross-resistance of HCV to ribavirin and other antiviral drugs that could have a mechanism of action similar to that of ribavirin, we tested the ability of a J6/JFH1 [11] HCV derived ribavirin resistant mutant, HCV-RR [12], to grow in the presence of the pyrimidine analog 5-Fluorouracil and the nucleoside analog 2′-C-Methylcytidine in Huh7D cells (a Huh7 cell derivative more permissive to HCV replication) [13]. 5-Fluorouracil is broadly used in the clinic to treat cancer [14] including HCV associated hepatocellular carcinoma [15]. 5-Fluorouracil displays mutagenic activity leading to viral extinction in different RNA viruses including LCMV, VSV, EMCV, and FMDV when grown in tissue cultured cells [16] and a similar lethal mutagenic effect has also been observed for ribavirin on several viruses including poliovirus [17], coxsackievirus B3 [18], FMDV [19], West Nile virus [20], GB virus B [21], and Hantaan virus [22]. It has been shown that ribavirin also has a mutagenic effect on HCV, increasing its mutation rate in cultured cells [23]–[26] and in vivo [25], [27]. Deep sequencing has recently revealed that ribavirin exerts mutagenic activity in chronic HCV infected patients by facilitating G to A and C to U nucleotide transitions [28]. 2′-C-Methylcytidine, the active component of the experimental anti-HCV pro-drug valopicitabine [29] [30], has been tested in HCV clinical trials and shown to be a potent HCV inhibitor in patients [31] [32]–[34] and chimpanzees [35]. 2′-C-Methylcytidine inhibited HCV RNA replication in the replicon assay and inhibited the HCV RNA polymerase *in vitro* in cell-free biochemical assays [36]. It has also been shown that ribavirin antagonizes the in vitro anti-HCV activity of 2′-C-Methylcytidine [37], suggesting an interaction between the two drug pathways.

In this study we show that an HCV mutant resistant to ribavirin is more resistant than parental J6/JFH1 to 5-Fluorouracil, but is not more resistant than parental J6/JFH1 to 2′-C-Methylcytidine. These results indicate that ribavirin resistant viruses could have elevated resistance to other inhibitors, highlighting the importance of combination drug selection for HCV treatment, and shedding light toward the understanding of the mechanism of action of ribavirin and HCV resistance to this drug.

In addition, the growth of HCV-RR in 5-Fluorouracil allowed us to select an HCV mutant resistant to 5-Fluorouracil that can replicate *in vitro* to moderate levels in the presence of concentrations as high as 3 µ/ml of 5-Fluorouracil and to parental levels in the absence of drug. The 5-Fluorouracil resistant virus accumulated multiple mutations distributed throughout the HCV genome.

## Results

### Growth of HCV in the Presence of 5-Fluorouracil

**Figure 1:**
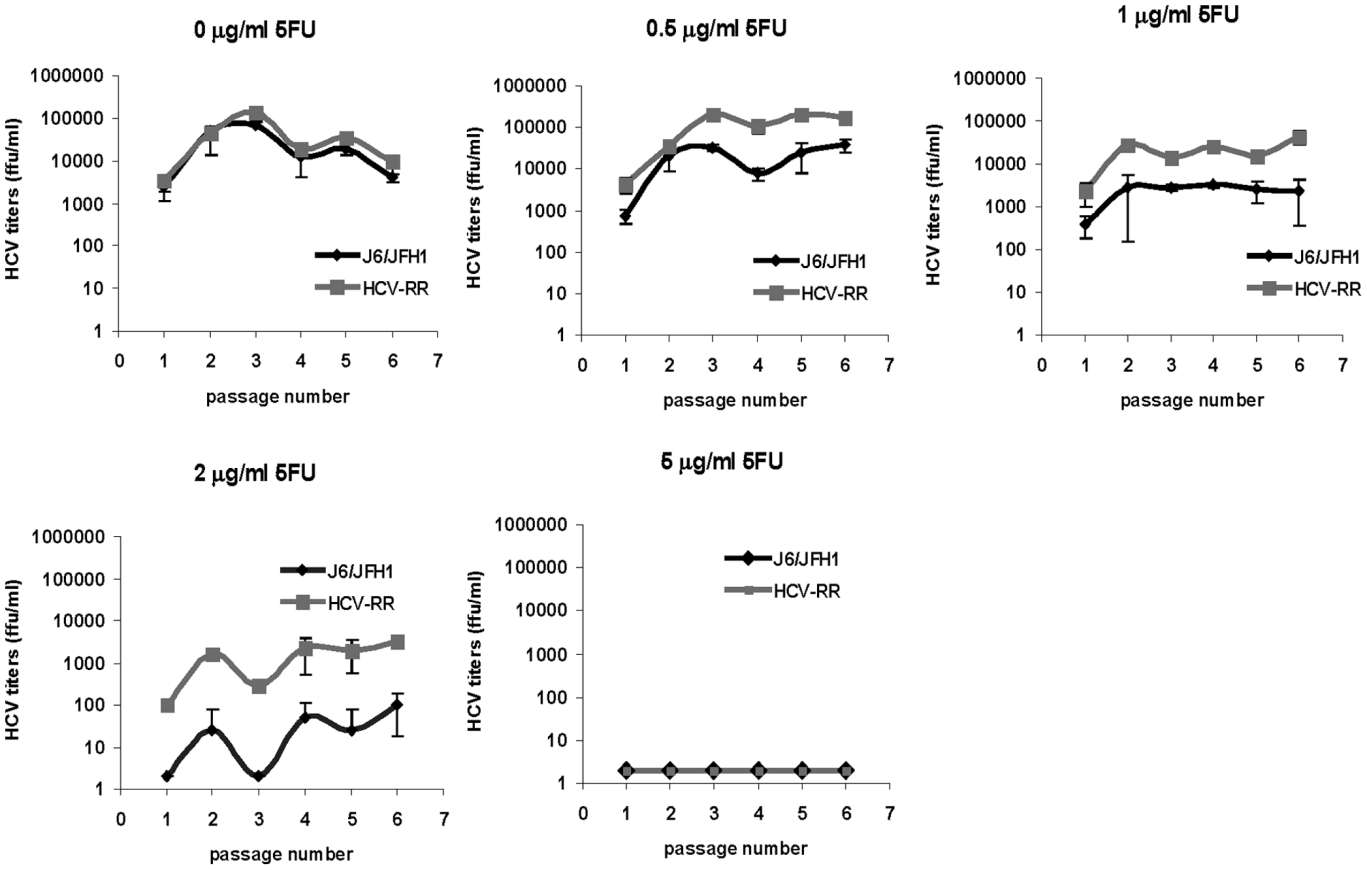
Growth of HCV in the presence of 5-Fluorouracil. J6/JFH1 and HCV-RR2 viruses were serially passaged in Huh7D cells in medium containing the indicated concentration of 5-Fluorouracil. At each passage HCV titers were obtained as described in the text. Titers are expressed as the mean number of foci of each of four replicates. Error bars represent the standard deviation.

In order to test the sensitivity of an HCV ribavirin resistant mutant to 5-Fluorouracil, parental J6/JFH1 [11] and J6/JFH1 derived HCV-RR2 [12] were grown in zero, 0.5, 1, 2, and 5 µg/ml of 5-Fluorouracil by serially passaging the viruses every 7 days in naïve Huh7D cells [13] as described in the materials and methods section. Virus growth was assessed by immunfluorscence (not shown) and by titration of virus present in the supernatants from each passage (Figure 1). Both viruses grew similarly in medium containing no 5-Fluorouracil. HCV-RR2 grew to higher titers than J6/JFH1 in medium containing 0.5, 1, and 2 µg/ml of 5-Fluorouracil. Neither virus survived a concentration of 5 µg/ml of 5-Fluorouracil. The experiment was repeated several times and similar results were obtained. The result indicates that HCV-RR2 is more resistant to 5-Fluorouracil than parental J6/JFH1.

### Growth of HCV in the Presence of 2′-C-Methylcytidine

**Figure 2:**
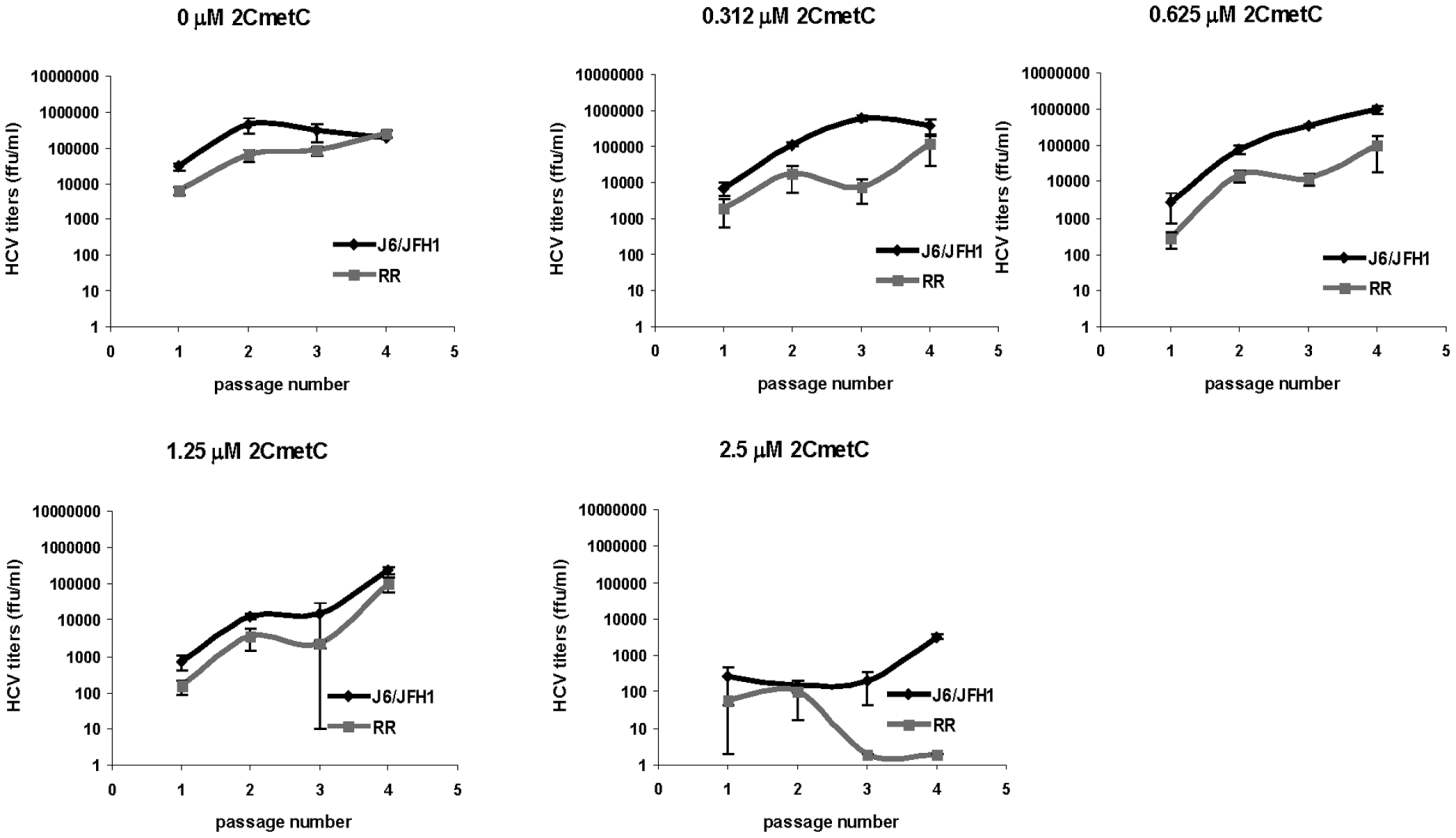
Growth of HCV in the presence of 2′-C-Methylcytidine. J6/JFH1 and HCV-RR2 viruses were serially passaged in Huh7D cells in medium containing the indicated concentration of 2′-C-Methylcytidine. At each passage HCV titers were obtained as described in the text. Titers are expressed as the mean number of foci of each of four replicates. Error bars represent the standard deviation.

In order to test sensitivity of an HCV ribavirin resistant mutant to 2′-C-Methylcytidine, parental J6/JFH1 and HCV-RR2 were grown in zero, 0.31, 0.62, 1.25, and 2.5 µM 2′-C-Methylcytidine as described above for 5-Fluorouracil. Virus growth was assessed by immunofluorescence (not shown) and by titration of virus present in the supernatants from each passage (Figure 2). Both viruses grew similarly in medium containing 0, 0.31, 0.62, and 1.25 µM of 2′-C-Methylcytidine. At a concentration of 2.5 µM of 2′-C-Methylcytidine, parental J6/JFH1 grew to titers of more than 10^2^ ffu/ml and 10^3^ ffu/ml by passage 3 and 4, while HCV-RR2 was extinguished after passage 2. This result indicates that HCV-RR2 is more sensitive to 2′-C-Methylcytidine than parental J6/JFH1 after passaging in medium containing 2.5 µM of 2′-C-Methylcytidine.

In another experiment, J6/JFH1 and HCV-RR2 were grown in the presence of no drug, 250 µM ribavirin, 1.5 µg/ml 5-Fluorouracil, or 1.25, 2.5 or 5 µM 2′-C-Methylcytidine (Figure S1). Both viruses grew in the presence of no drug. HCV-RR2 grew in 250 µM ribavirin, while J6/JFH1 did not. HCV-RR2 grew to titers that were more than 1 log higher than J6/JFH1 in 1.5 µg/ml 5-Fluorouracil. J6/JFH1 grew in the presence of 1.25 µM 2′-C-Methylcytidine, while HCV-RR2 did not. None of the viruses grew in 2.5 and 5 µM 2′-C-Methylcytidine. This result confirms the differential sensitivity of J6/JFH1 and HCV-RR2 to 5-Fluorouracil and 2′-C-Methylcytidine.

**Figure 3:**
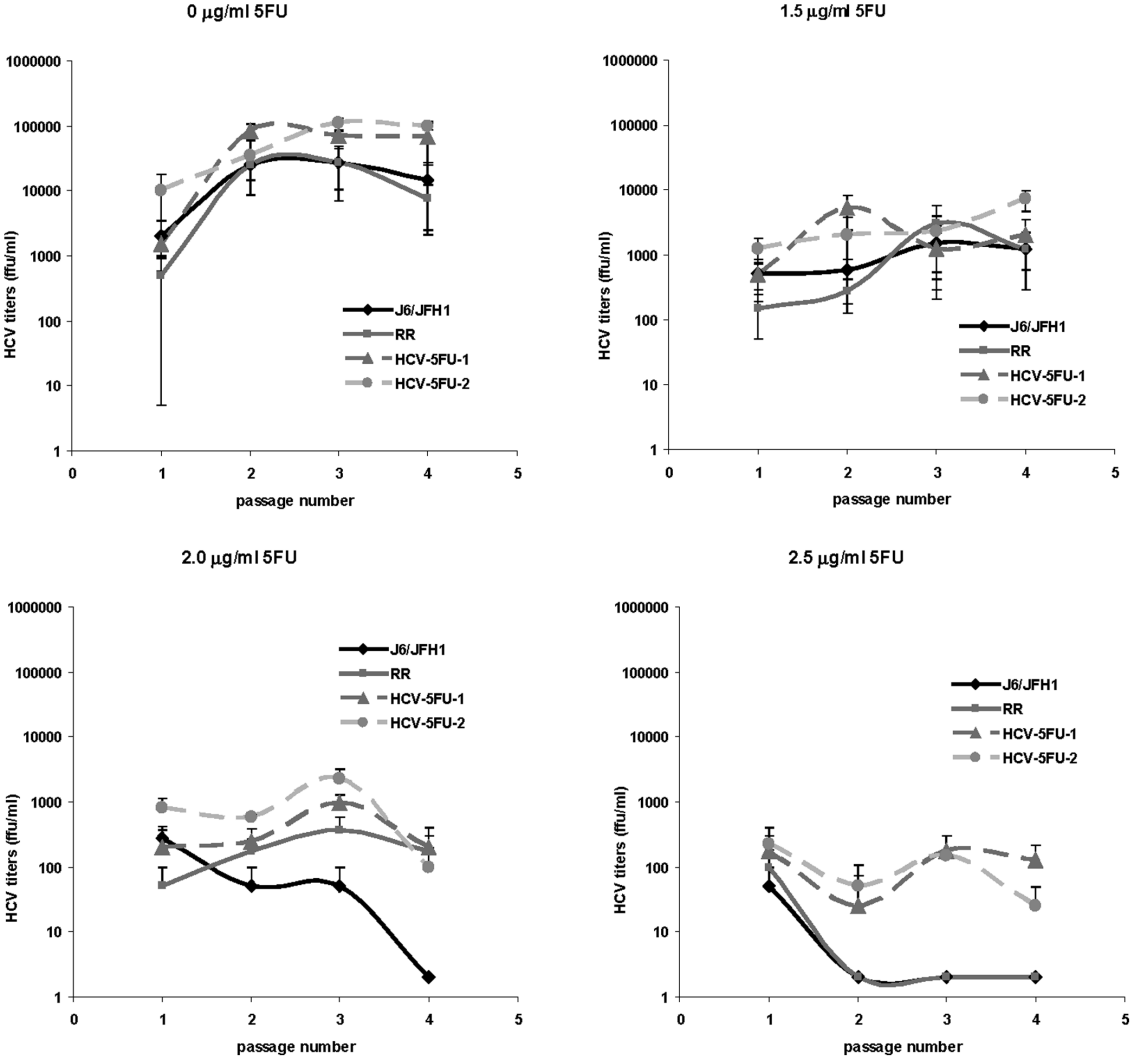
HCV recovered from 5-Fluorouracil treated cells is resistant to 5-Fluorouracil. J6/JFH1, HCV-RR2, HCV-5FU-1-P6, and HCV-5FU-2-P6 were serially passaged in Huh7D cells in medium containing the indicated concentration of 5-Fluorouracil. At each passage HCV titers were obtained as described in the text. Titers are expressed as the mean number of foci of each of four replicates. Error bars represent the standard deviation.

We note variations in the growth of the viruses among experiments. For example, J6/JFH1 and HCV-RR2 grew to a lesser extent in 1.25 µM 2′-C-Methylcytidine in the experiment shown in Figure S1 when compared to the growth attained in the experiment shown in Figure 2. Similarly, J6/JFH1 and HCV-RR2 grew to a lesser extent in 2 µg/ml 5-Fluorouracil in the experiment shown in Figure 3 (see below) when compared to the growth attained in the experiment shown in Figure 1. We don’t know at this time the nature of these variations.

### Virus Recovered from 5-Fluorouracil Treated Cells is Resistant to 5-Fluorouracil

The enhanced growth of HCV-RR2 in 5-Fluorouracil (Figure 1) prompted us to obtain a 5-Fluorouracil HCV resistant virus. To that end, we subjected HCV-RR2 to six passages of 7 days each in Huh7D cells treated with 2.5 µg/ml of 5-Fluorouracil followed by a) one passage of 7 days in medium containing no 5-Fluorouracil to obtain HCV-5FU-1 or b) two passages (of 3 and 7 days) in medium containing no 5-Fluorouracil to obtain HCV-5FU-2. These last passages in medium containing no drug were performed in order to increase the titer of the viruses. Viruses were titrated to 2.2×10^3^ ffu/ml and 2.5×10^3^ ffu/ml respectively. In order to test whether the HCV-5FU obtained viruses were truly resistant to 5-Fluorouracil, J6/JFH1, HCV-RR2, HCV-5FU-1 and HCV-5FU-2 were grown in 0, 1.5, 2 and 2.5 µg/ml 5-Fluorouracil by serially passaging the viruses every 7 days in naïve Huh7D cells as described for the experiment shown in Figure 1. Virus growth was assessed by immunofluorescence (not shown) and by titration of the supernatants from each passage (Figure 3). All viruses grew in medium containing 0 or 1.5 µg/ml 5-Fluorouracil. HCV-RR2, HCV-5FU-1 and HCV-5FU-2 grew in 2 µg/ml 5-Fluorouracil, while J6/JFH1 was extinct by passage 4. Only HCV-5FU-1 and HCV-5FU-2 grew in 2.5 µg/ml 5-Fluorouracil. This result indicates that viruses recovered from 5-Fluorouracil treated cells were more resistant to 5-Fluorouracil than parental HCV-RR2.

**Figure 4:**
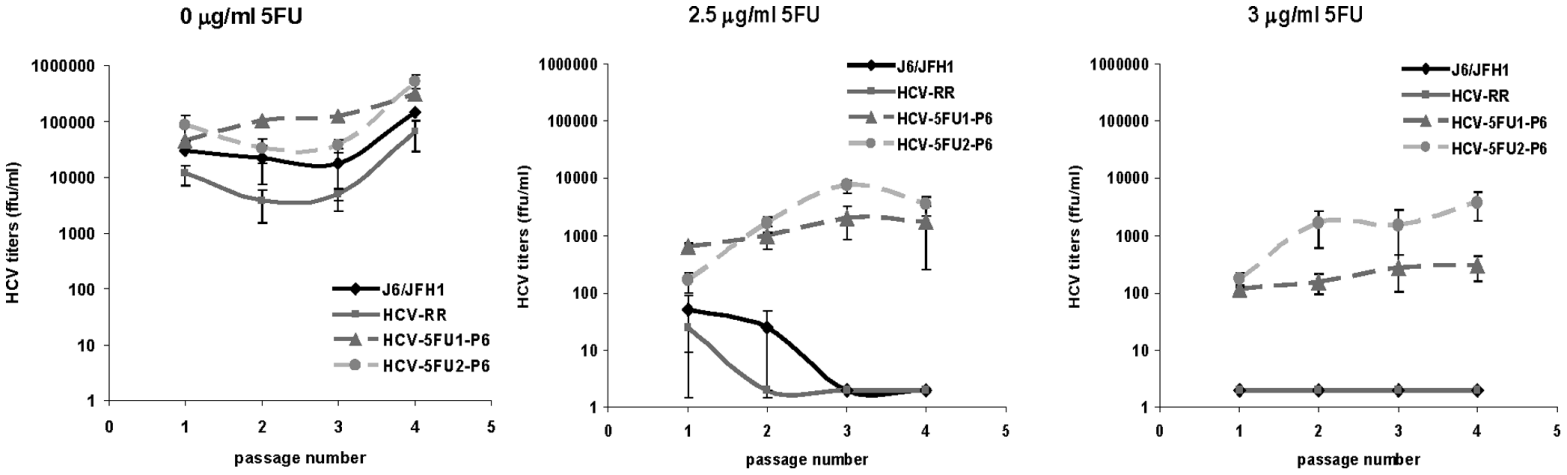
Further passage of HCV in 5-Fluorouracil yields viruses resistant to 3 µg/ml concentration of 5-Fluorouracil. J6/JFH1, HCV-RR2, HCV-5FU-1-P6, and HCV-5FU-2-P6 were serially passaged in Huh7D cells in medium containing the indicated concentration of 5-Fluorouracil. At each passage HCV titers were obtained as described in the text. Titers are expressed as the mean number of foci of each of four replicates. Error bars represent the standard deviation.

HCV-5FU-1 and HCV-5FU-2 that were passed six times in 2.5 µg/ml 5-Fluorouracil in the experiment shown in Figure 3 (designated HCV-5FU-1-P6 and HCV-5FU-2-P6) were further tested for their resistance to 2.5 µg/ml and 3 µg/ml 5-Fluorouracil in Huh7D cells. All viruses grew in the absence of 5-Fluorouracil. As seen for HCV-5FU-1 and HCV-5FU-2, HCV-5FU-1-P6 and HCV-5FU-2-P6 grew in 2.5 µg/ml 5-Fluorouracil achieving titers higher than 10^3^ ffu/ml, while J6/JFH1 and HCV-RR2 did not grow. Furthermore, HCV-5FU-1-P6 and HCV-5FU-2-P6 grew in 3 µg/ml 5-Fluorouracil achieving titers higher than 10^2^ and 10^3^ ffu/ml respectively, while J6/JFH1 and HCV-RR2 did not grow (Figure 4). We conclude that we isolated viruses that are resistant to concentrations as high as 3 µg/ml 5-Fluorouracil. HCV-5FU viruses grow to at least parental (J6/JFH1 and HCV-RR2) levels in the absence of the drug (Figure 4) and are still resistant to ribavirin (not shown).

### Kinetics of Virus Growth in 2 and 3 µM 5-Fluorouracil for One Week

**Figure 5:**
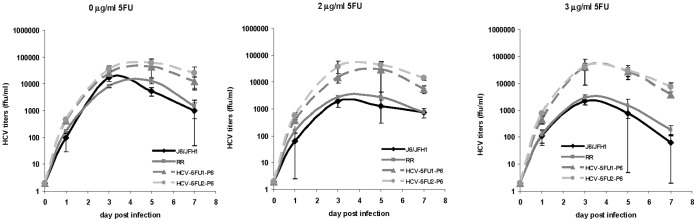
Growth of HCV in 2 and 3 µg/ml concentration of 5-Fluorouracil for one week. Huh7D cells were mock infected or infected with J6/JFH1, HCV-RR2, HCV-5FU-1-P6, and HCV-5FU-2-P6 at a m.o.i. of 0.01. After 6 hours, cells were washed with growth medium three times and left in a concentration of zero, 2, or 3 µg/ml of 5-Fluorouracil. 300 µl of each supernatant were collected at the indicated time points and frozen at −70°C. Wells were supplemented with 300 ul of medium containing corresponding concentration of 5-Fluorouracil. Virus was titered as described in the text. Titers are expressed as the mean number of foci of each of four replicates. Error bars represent the standard deviation.

We studied the 5-Fluorouracil resistant phenotype of HCV-5FU-1-P6 and HCV-5FU-2-P6 by determining the kinetics of the growth of J6/JFH1, HCV-RR2, HCV-5FU-1-P6 and HCV-5FU-2-P6 in a concentration of 0, 2, and 3 µg/ml of 5-Fluorouracil for one week as described in the material and methods section. By days 5 and 7, HCV-5FU-1-P6 and HCV-5FU-2-P6 reached titers that were more than 1 log higher than J6/JFH1 and HCV-RR2 in medium containing concentrations of 2 and 3 µg/ml of 5-Fluorouracil (Figure 5). Interestingly, HCV-5FU-1-P6 and HCV-5FU-2-P6 grew to slightly higher titers in medium containing no 5-Fluorouracil when compared to J6/JFH1 and parental HCV-RR2 (see Figures 3 and 4). The result confirms the resistance of HCV-5FU-1-P6 and HCV-5FU-2-P6 viruses to 5-Fluorouracil.

### Kinetics of Virus Growth in Different Concentrations of 2′-C-Methylcytidine for ′

**Figure 6:**
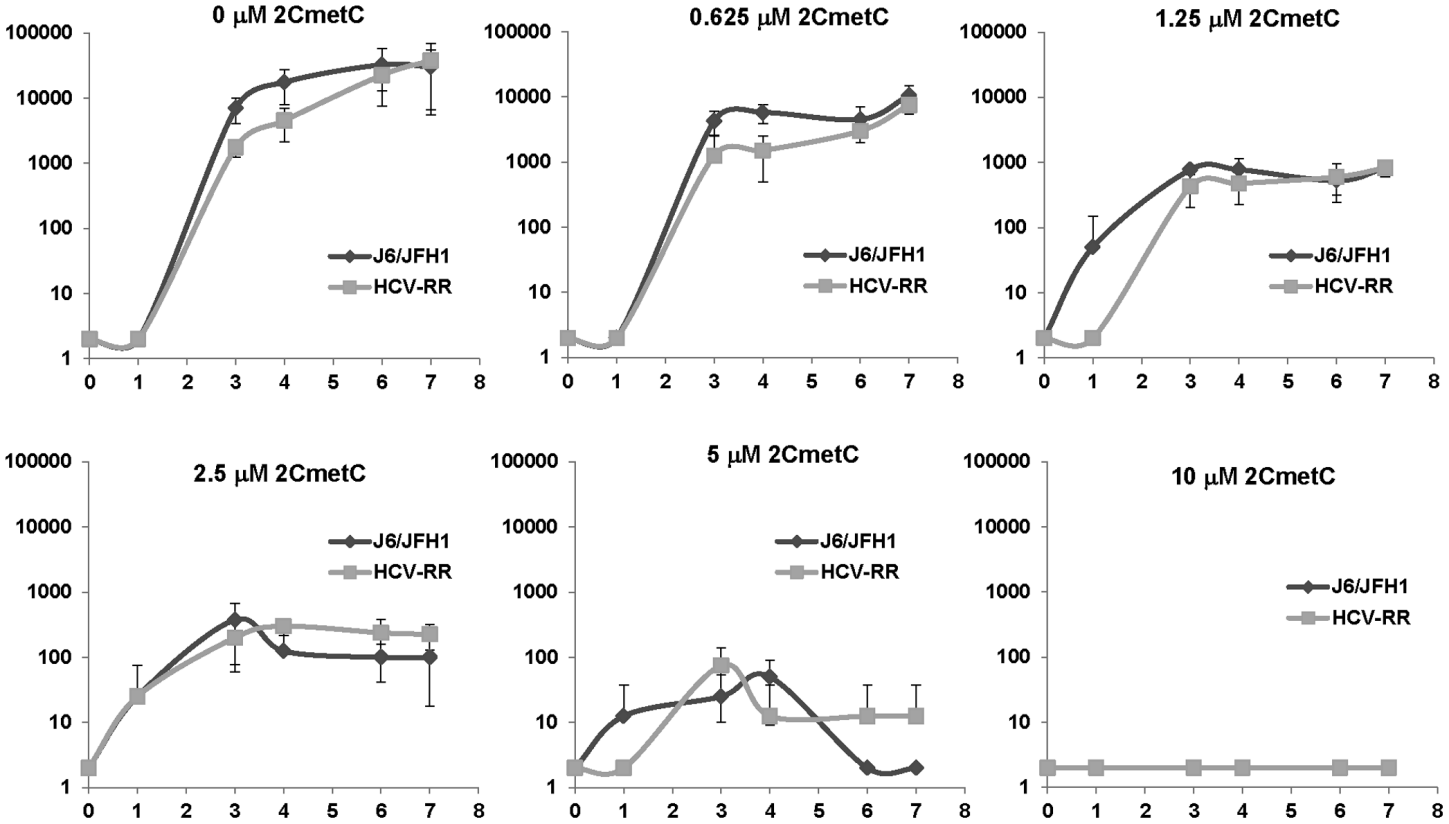
Growth of HCV in different concentrations of 2′-C-Methylcytidine for one week. Huh7D cells were mock infected or infected with J6/JFH1 and HCV-RR2 at a m.o.i. of 0.01. After 6 hours, cells were washed with growth medium three times and left in a concentration of zero, 0.625, 1.25, 2.5, 5, and or 10 µM of 2′-C-Methylcytidine. 150 µl of each supernatant were collected at the indicated time points and frozen at −70°C. Wells were supplemented with 150 ul of medium containing corresponding concentration of 2′-C-Methylcytidine. Virus was titered as described in the text. Titers are expressed as the mean number of foci of each of four replicates. Error bars represent the standard deviation.

We also studied the kinetics of the growth of J6/JFH1 and HCV-RR2 in concentrations of 0, 0.625, 1.25, 2.5, 5, and 10 µM of 2′-C-Methylcytidine for one week as described in the material and methods section. Similar growth curves were observed for J6/FH1 and HCV-RR2 (Figure 6). The result indicates that both viruses have a similar sensitivity to 2′-C-Methylcytidine by passage one. At later passages, J6/JFH1 seems to have a slight advantage over HCV-RR2 (Figure 2 and Figure S1).

### Virus Resistant to 5-Fluorouracil ′and Non-synonymous Mutations

In order to find out if 5-Fluorouracil resistant viruses acquired mutations, viral RNA from HCV-5FU-1-P6 and HCV-5FU-2-P6 was extracted, reverse transcribed, amplified by PCR, and sequenced. We obtained the sequence for HCV-5FU-1-P6 from nucleotide 80 through nucleotide 9460. In the coding region, HCV-5FU-1-P6 acquired 33 nucleotide substitutions (relative to HCV-RR2), 10 of which encoded amino-acid changes that were located in the core, E1, E2, P7, NS5a, and NS5b proteins (Table 1). HCV-5FU-1-P6 maintained all 38 mutations present in HCV-RR2 relative to parental J6/JFH1 [12]. No mutations were found in the non-coding regions. HCV-5FU-2-P6 was partially sequenced and showed a sequence that was identical to the HCV-5FU-1-P6 sequence.

**Table 1:** Mutations observed in HCV resistant to 5-Fluorouracil.

Nucleotide position*	Nucleotide inHCV-RR2	Nucleotide inHCV-5FU-1-P6	Protein	Amino-acid position* &	Amino-acid in HCV-RR2	Amino-acid in HCV-5FU-1-P6
593	C	T	CORE			
733	C	T		133	L	F
737	C	T				
1189	G	A	E1			
1349	G	A	E2	337	A	T
1480	G	A		405	M	R
1554	T	G				
1609	C	T				
1711	C	T				
1765	G	A				
1876	C	T				
2598	A	G	P7	753	E	G
2608	C	T				
3034	G	A	NS2			
3736	C	T	NS3			
3871	G	A				
4129#	T	G/T				
5575	G	A	NS4b			
5656	C	T				
5803	G	T				
5812	C	T				
6043	C	T				
6076	G	A				
6578#	A	G/A	NS5a	2080	T	A/T
7104	T	C		2255	F	S
7143	C	T		2268	P	L
7217#	T	C/T		2293	Y	H/Y
7387	C	T		2397	G	R
7459#	C	G/C				
7504	C	T				
7529	G	A				
8254	C	T	NS5b			
8483	A	G		2715	T	A

* nomenclature is according to the JFH1 sequence, accession number AB047639.

& Only amino-acids where a substitution in HCV-5FU-1-P6 relative to HCV-RR2 was found are indicated.

# Sequence at positions 4129, 6578, 7217, and 7459 indicates a mix nucleotides.

We analyzed each of the ten positions where nucleotide substitutions leading to amino-acid changes were found in HCV-5FU-1-P6 by sequencing viruses recovered after passage 1, 2, 3, and 4 of HCV-RR2 in 2.5 µg/ml 5-Fluorouracil, and by sequencing HCV-5FU-1 (Table 2). All the mutations found in HCV-5FU-1-P6 were already found in HCV-5FU-1, with the exception of T2080T/A and Y2293Y/H. Some of the mutations found in HCV-5FU-1 (and HCV-5FU-1-P6) were present at early passages of HCV-RR2 in 5-Fluorouracil, as L133F in the core protein, M405R in E2, and P2268L and G2397R in NS5a. Mutation T2715A in NS5b appeared after passage 3.

**Table 2:** Nucleotide and deduced amino acid changes in early passages of HCV-RR2 in 5-Fluorouracil at positions found mutated in HCV-5FU-1-P6.

Nucleotide*	HCV-RR2	P1	P2	P3	P4	HCV-5FU-1	HCV-5FU-1-P6	amino acid change*
737	C	C/T	C/T	C/T	T	T	T	L133F (core)
1349	G	G	G>A	A/G	A	A	A	A337T (E1)
1554	T	T	T/G	G	G	G	G	M405R (E2)
2598	A	A	A	G>A	G	G	G	E753G (P7)
6578	A	A	ND	A	A	A	G/A	T2080T/A (NS5a)
7104	T	T	T	C	C	C	C	F2255S (NS5a)
7143	C	C>T	T/C	T	T	T	T	P2268L (NS5a)
7217	T	T	T	T	T	T	T/C	Y2293Y/H (NS5a)
7529	G	G	G/A	G/A	G/A	A	A	G2397R (NS5a)
8483	A	A	A	A	ND	G	G	T2715A (NS5b)

* nomenclature is according to the JFH1 sequence, accession number AB047639.

ND: not determined.

## Discussion

In this study we compared the growth of an HCV ribavirin resistant mutant (HCV-RR2) to the growth of its parental J6/JFH1 virus in the presence of two antiviral drugs: the mutagenic pyrimidine 5-Fluorouracil and the nucleoside analog 2′-C-Methylcytidine. We show that HCV resistant to ribavirin is more resistant to 5-Fluorouracil but is not more resistant to 2′-C-Methylcytidine (Figures 1 and 2) than it parental virus J6/JFH1. By passaging HCV-RR2 in 5-Fluorouracil we selected HCV-RR2 resistant to 5-Fluorouracil (HCV-5FU). We confirmed the 5-Fluorouracil resistant phenotype of HCV-5FU viruses by infecting naïve cells and showing that they can grow even in a concentration of 3 µg/ml 5-Fluorouracil (Figures 3, 4, and 5). HCV-5FU resistant to 5-Fluorouracil acquired synonymous and non-synonymous mutations that were distributed all along the genome (Table 1).

The mechanism of action of ribavirin against HCV *in vitro* and *in vivo* and the mechanism of resistance to ribavirin by HCV ribavirin resistant mutants has not been completely elucidated. The differential sensitivity observed for HCV-RR2 to 5-Fluorouracil and 2′-C-Methylcytidine when compared to parental J6/JFH1 virus indicates that mechanisms and/or viral RNA sequences implicated in the antiviral activity of these drugs could be involved in mechanism and/or viral RNA sequences of the antiviral activity of ribavirin. This is supported by the fact that 5-Fluorouracil is a pyrimidine analog and 2′-C-Methylcytidine and ribavirin are both nucleoside analogs. Given the nature of these drugs, it is tempting to speculate that ribavirin acts on HCV at the RNA replication level, as it has been shown for 2′-C-Methylcytidine [36]. We previously showed that an HCV ribavirin resistant mutant has mutations in different positions of its genome including the RNA dependent RNA polymerase [12], and others found that the RNA dependent RNA polymerase from HCV can use ribavirin triphosphate as a nucleotide substrate. Once ribavirin monophosphate has been incorporated in the nascent chain, it can reduce or even block RNA elongation [38], [39]. As noted above, mutagenic activity of ribavirin on HCV has been observed *in vivo* and *in vitro* [23]–[26] [25], [27] [28].

We found that HCV-RR2 was more resistant to the mutagen 5-Fluorouracil than its parental J6/JFH1. This indicates that determinants conferring resistance to ribavirin also confer resistance to 5-Fluorouracil and could confer resistance to other antiviral drugs. Cross resistance in HCV has been observed by other investigators. As an example, in the replicon system, an HCV mutant resistant to 2′-C-Methylcytidine showed cross-resistance to the nucleoside analog 2′-C-Methyladenosine but not to the nucleoside analog 4′-Azidocytidine (R1479), interferon α-2a, or to non-nucleoside HCV polymerase inhibitors [36]. These observations in cultured cells could be also relevant *in vivo*. Therefore, careful considerations should be made in the clinic when selecting combined or sequential drug treatments. Given the observed efficacy of the inclusion of ribavirin in interferon free regimens in the treatment of chronic HCV, cross resistance of HCV to ribavirin and to other antiviral drugs could be of major importance.

In FMDV, a single point mutation (M296I) confers resistance to ribavirin [40]. This mutant was as sensitive as wild type FMDV to 5-fluorouracil when administered in combination with guanidine hydrochloride, indicating that mutation M296I did not confer a significant cross-resistance to 5-fluorouracil [41]. These and our results indicate that different viruses evolve to generate phenotypicaly different mutants to escape the action of antiviral drugs. Of note, the concentrations of ribavirin (5 mM) and 5-Fluorouracil (200 or 500 µg/ml) for the treatment of BHK-21 cells used by Perales and colleagues are between 1 and 3 orders of magnitude higher than those used in our study.

We isolated an HCV mutant resistant to 5-Fluorouracil (HCV-5FU). To our knowledge, no 5-Fluorouracil resistant viruses, including HCV, have been previously reported. When compared to its parental HCV-RR2, HCV-5FU acquired 33 mutations in the coding region, 10 of which encoded amino-acid changes. Of note, all the 38 mutations acquired by HCV-RR2 when compared to its parental J6/JFH1 [12] were maintained in HCV-5FU even after several passages for selection (see above, results, and Table 1). We don’t know which mutation/s confer resistance to 5-Fluorouracil. Six of the ten non-synonymous mutations acquired by HCV-5FU are located in non-structural proteins NS5a and NS5b (Table 1). Mutation A8483G encodes a change from threonine to alanine at amino-acid 273 of NS5b, which, according to its crystal structure, is located in the finger domain [42], [43]. HCV-RR2 acquired mutation G7710A which encodes a mutation at amino-acid 15 of NS5b, also located in the finger domain [12]. Mutations in the finger domain have been identified in ribavirin resistant mutants of poliovirus [44], [45]. Five other non-synonymous mutations observed in HCV-5FU are located in positions corresponding to domains I (one mutation), II (three mutations) and III (one mutation) of NS5a [46]. Although the role of NS5a has not been completely elucidated, it is known that NS5a is essential for HCV replication, interacts with other HCV and cellular proteins forming multiprotein replication complexes [47]–[49] and has been associated with sensitivity to interferon [50]. Therefore, NS5a mutations carried by HCV-5FU may alter its interaction with other viral proteins as NS5b, critical for viral replication.

## Conclusion

In this report, we show that a ribavirin resistant mutant of HCV has differential sensitivity to other antiviral drugs when compared to its parental virus. This suggests that mutations that are responsible for HCV resistance to ribavirin can be involved in the sensitivity to other drugs, implying that common antiviral mechanisms and common mechanisms of defense could be used by and against different drugs. This could be clinically important for drug selection, since an interferon-free regimen containing ribavirin for the treatment of HCV infections seems currently plausible. We isolated a mutant resistant to the potent mutagen 5-Fluorouracil. This mutant when grown without 5-Fluorouracil can replicate to parental levels. Analysis of the mutations responsible for the 5-Fluorouracil resistance phenotype may aid in understanding the mechanism of action of 5-Fluorouracil and other antivirals against HCV.

## Methods

### Cells

Huh7D cells, a highly permissive clone derived from Huh7 cells [13] were grown in DMEM (Gibco) supplemented with 10% bovine calf serum (Atlanta Biologicals), L-glutamine (Gibco), penicillin, and streptomycin (Gibco).

### Viruses

Virus J6/JFH1 was obtained from plasmid pFL- J6/JFH1 [11] (a plasmid coding for full length J6/JFH1 virus, kindly provided by Dr Charles Rice) as previously described [13]. HCV-RR2 virus was previously described [12].

### Antibodies

Monoclonal antibody 6G7 directed to the HCV core protein was kindly provided by Henry H. Hsu and Harry B. Greenberg (Stanford University, Palo Alto Veterans Administration Medical Center, Palo Alto, CA) [51].

### Infection of Huh7D Cells with J6/JFH1, HCV-RR, and HCV-5FU Viruses and Treatment with 5-Fluorouracil or 2′-C-Methylcytidine

Huh7D cells grown in 48-well plates were mock infected or infected with the indicated viruses at a moi of 0.01. At 5 to 7 hours post infection, medium was replaced with 500 µl of medium containing the indicated amount of 5-Fluorouracil (Sigma) or 2′-C-Methylcytidine (USBiological). At 7 days post infection, 200 µl of the supernatants were used to inoculate naïve Huh7D cells that were seeded the day before, and at 5 to 7 hours post infection medium was replaced with 500 µl of medium containing the corresponding concentration of corresponding drug. The rest of the supernatants were stored at −70°C. This procedure was repeated for the indicated number of passages. HCV antigen was detected in the remaining monolayers by immunofluorescence and HCV titers were obtained from the supernatants as described below.

### Growth of HCV in 5-Fluorouracil or 2′-C-Methylcytidine for One Week

Huh7D cells grown in a 12 well plate were mock infected or infected with J6/JFH1, HCV-RR2, HCV-5FU-1-P6, or HCV-5FU-2-P6 at a moi of 0.01. At 6 hours post infection, cells were washed three times with medium. After final wash, medium was replaced with 1.5 ml of medium containing a final concentration of zero, 2, or 3 µg/ml of 5-Fluorouracil or 0.625, 1.25, 2.5, 5, or 10 µM concentration of 2′-C-Methylcytidine. At different days post infection, supernatants were taken from each well and stored at −70°C. Wells were supplemented with medium containing corresponding concentration of 5-Fluorouracil or 2′-C-Methylcytidine. Virus from each time point was titered as described below.

### Titration of Viruses

Monolayers of Huh7D cells grown in 96 well plates were infected with 100 µl of 10-fold serial dilutions of the corresponding virus (in quadruplicates). At three days post infection, viral antigen was detected by immunofluorescence as described below. Foci were counted and titers were expressed as the mean number of foci of each of the four replicates +/− the standard deviation.

### Detection of HCV Antigen by Immunofluorescence

Infected cells were fixed with methanol, washed with 1×PBS, blocked with a solution containing 1% BSA and 0.2% non-fat milk in 1×PBS, washed with 1×PBS, treated with a 1∶400 dilution of monoclonal antibody 6G7 in 0.05% tween 20 in 1×PBS, washed with 1×PBS, stained with FITC-conjugated goat anti-mouse antibody (KPL), washed with 1×PBS, and observed in the microscope with UV light. The percentage of positive cells was determined by dividing the estimated amount of positive cells over the total number of cells per well.

### Sequencing of Ribavirin Resistant Viruses

Viral RNA was extracted from virus stocks using Trizol reagent as recommended by the manufacturer (Invitrogen). cDNA was synthesized using SuperScript III reverse transcriptase and random primers (Invitrogen). PCR amplification of the HCV genome was performed using the Expand High Fidelity PCR system (Roche) as recommended by the manufacturer and the following sets of primers:

2a40+ (5′-atgaatcactcccctgtgag-3′) and 2a1260- (5′-gagcaattgcagtcttggac-3′),

2a1101+ (5′-TCACGCAGGGCTTGCGGACG-3′) and 2a2690- (5′- CCTTGATGTACCAAGCAGCC-3′), 2a2431+ (5′-CCAAAACATCGTGGACGTAC-3′) and 2a3980- (5′-AAGTGGGAGACCTTGTAACA-3′), 2a3721+ (5′-CAAGTGTGGAGCCGTCGACC-3′) and 2a5051+ (5′-CACATAGACGCCCACTTCCT-3′) or 2a6320- (5′-CTGTCAAGATGGTGCAAACC, 2a6181+ (5′-TGTGACCCAACTACTTGGCT-3′) and negNS5b15mut (5′-CTCTTCGGGGTTACAGGGAGTTATTAGAGCCCC-3′),

2a6781+ (5′-TGAGGTCTCGTTCTGCGTTG-3′) and 2a7860- (5′-TCATAATGGGCGTCGAGCAC-3′), 2a7141+ (5′-GCCCTCAATACCATCGGAGT-3′) and 2a8250- (5′-TACTGGAAGCCATAGGAAGC-3′).

2a8041+ (5′-CCTCCTGGAAGACCCACAAA-3′) and 2a9480- (5′- GAACAGTTAGCTATGGAGTG-3′).

PCR products were run in agarose gels and purified using gene-elute agarose gel columns (Sigma) or the QIAquick PCR purification Kit (Qiagen) and sequenced using the BigDye terminator v3.1 cycle-sequencing kit (Applied Biosystems) and the 3130×l Genetic analyzer (Applied Biosystems). In addition to the oligos used for PCR, we used the following oligos for sequencing: 2a4280- (5′-AGGCGCCGCTAGCGCAGCCC-3′), 2101+ (5′-GGACTGTTTTAGGAAGCATC-3′), 2a2400- (5′-GGCAGGTCCGAGTAAGAGCA-3′), 2a3560- (5′-GGAAGGACTGAGAGACTGTG-3′), 2a3671+ (5′-GACTTGGTAGGCTGGCCCAG-3′), 2a4340- (5′-CGATGCCGAGAATGGAGGTA-3′), 2a4431+ (5′-CCGATATAGAAGAGGTAGGC-3′), 2a8521+ (5′-AACCACTAGCATGGGTAACA-3′), 2a9001+ (5′- TGAGATGTATGGATCAGTAT -3′), and 2a9040+ (5′- CTTCCAGCCATAATTGAGAG -3′).

## Supporting Information

Figure S1 Growth of HCV in the presence of different drugs. J6/JFH1 and HCV-RR2 viruses were serially passaged in Huh7D cells in medium containing the indicated concentration of the indicated drugs. At each passage HCV titers were obtained as described in the text. Titers are expressed as the mean number of foci of each of four replicates. Error bars represent the standard deviation.
(TIFF)
