# Supplementary figures and images for: Sensitivity of a Ribavirin Resistant Mutant of Hepatitis C Virus to Other Antiviral Drugs
Source: PLoS One. 2013 Sep 5;8(9):e74027. doi: 10.1371/journal.pone.0074027 (PMC3764029; doi:10.1371/journal.pone.0074027)

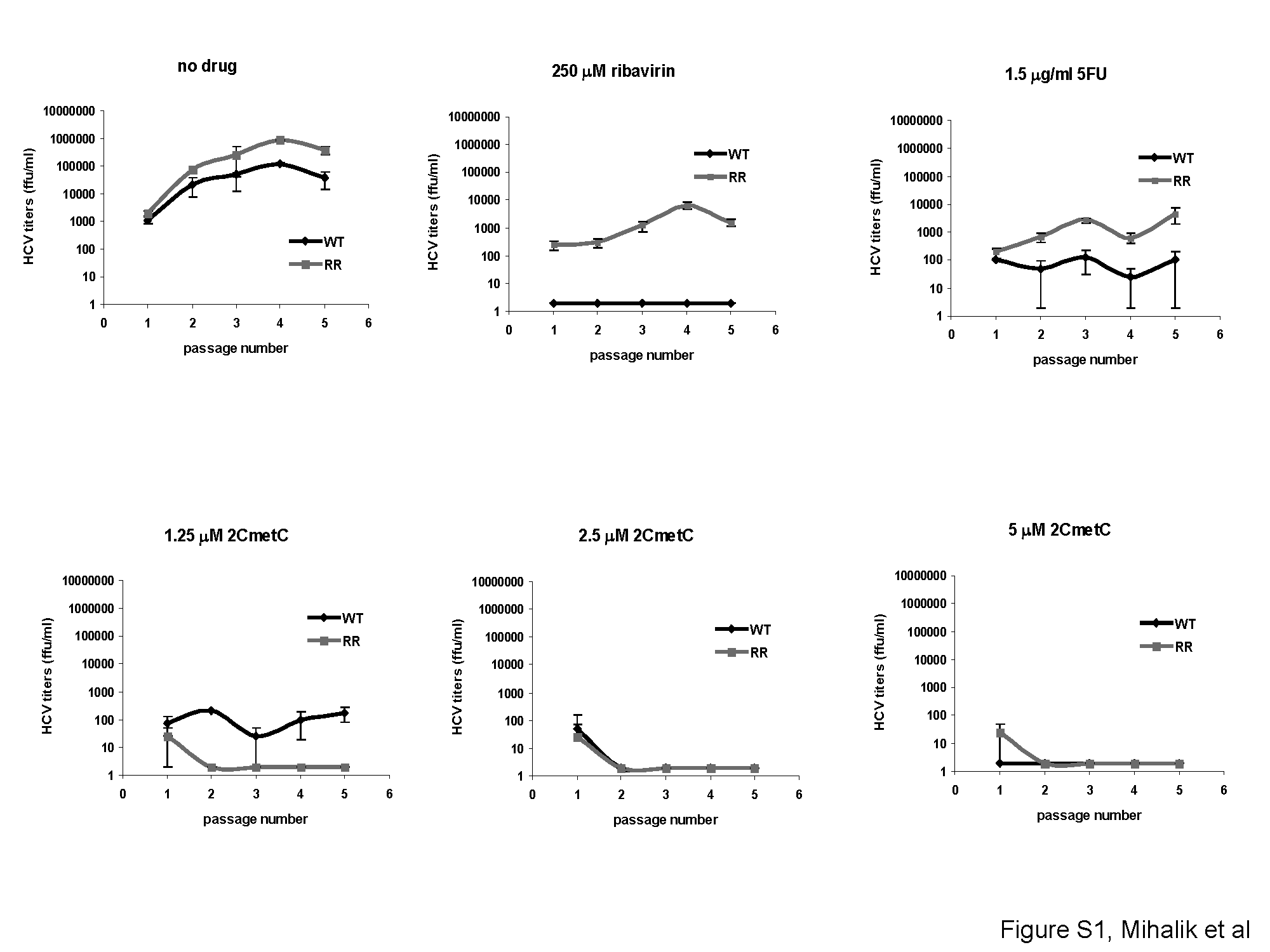

Supplement: Figure S1 — Growth of HCV in the presence of different drugs. J6/JFH1 and HCV-RR2 viruses were serially passaged in Huh7D cells in medium containing the indicated concentration of the indicated drugs. At each passage HCV titers were obtained as described in the text. Titers are expressed as the mean number of foci of each of four replicates. Error bars represent the standard deviation. (TIFF) [file pone.0074027.s001.tiff]
